# The acoustic bases of human voice identity processing in dogs

**DOI:** 10.1007/s10071-022-01601-z

**Published:** 2022-02-10

**Authors:** Anna Gábor, Noémi Kaszás, Tamás Faragó, Paula Pérez Fraga, Melinda Lovas, Attila Andics

**Affiliations:** 1 MTA-ELTE “Lendület” Neuroethology of Communication Research Group, Hungarian Academy of Sciences - Eötvös Loránd University, 1/C Pázmány Péter sétány, Budapest, 1117 Hungary; 2 Department of Ethology, Eötvös Loránd University, 1/C Pázmány Péter sétány, Budapest, 1117 Hungary

**Keywords:** Dog, Speaker-sensitivity, Interspecific voice discrimination, Acoustics

## Abstract

Speech carries identity-diagnostic acoustic cues that help individuals recognize each other during vocal–social interactions. In humans, fundamental frequency, formant dispersion and harmonics-to-noise ratio serve as characteristics along which speakers can be reliably separated. The ability to infer a speaker’s identity is also adaptive for members of other species (like companion animals) for whom humans (as owners) are relevant. The acoustic bases of speaker recognition in non-humans are unknown. Here, we tested whether dogs can recognize their owner’s voice and whether they rely on the same acoustic parameters for such recognition as humans use to discriminate speakers. Stimuli were pre-recorded sentences spoken by the owner and control persons, played through loudspeakers placed behind two non-transparent screens (with each screen hiding a person). We investigated the association between acoustic distance of speakers (examined along several dimensions relevant in intraspecific voice identification) and dogs’ behavior. Dogs chose their owner’s voice more often than that of control persons’, suggesting that they can identify it. Choosing success and time spent looking in the direction of the owner’s voice were positively associated, showing that looking time is an index of the ease of choice. Acoustic distance of speakers in mean fundamental frequency and jitter were positively associated with looking time, indicating that the shorter the acoustic distance between speakers with regard to these parameters, the harder the decision. So, dogs use these cues to discriminate their owner’s voice from unfamiliar voices. These findings reveal that dogs use some but probably not all acoustic parameters that humans use to identify speakers. Although dogs can detect fine changes in speech, their perceptual system may not be fully attuned to identity-diagnostic cues in the human voice.

**Supplementary Information:**

The online version contains supplementary material available at 10.1007/s10071-022-01601-z.

## Introduction

In vocal social species, the ability to recognize individuals based on voice is one of the most crucial functions of the auditory system. During voice identity recognition, individuals rely on the acoustic structure of vocalizations. Individual voices can be seen as points in a multidimensional ‘voice space’ in which vocalizers are separated along numerous acoustic dimensions (Baumann and Belin 2010). Although a wider variety of such dimensions would be suitable to differentiate voices, the perceptual system usually outbalances some of them during recognition (Kriengwatana et al. 2014). Voices closer to each other along perceptually important voice identity markers are typically harder to discriminate (Baumann and Belin 2010; Latinus and Belin 2011; Latinus et al. 2013). As voice identity recognition is more relevant in intraspecific interactions, research of its acoustic foundations concentrates on voices of conspecifics. In some special cases (e.g., in dog–human relationships), however, voice-based recognition of heterospecific individuals could be advantageous. While some studies indicate that dogs can associate voice identity information to speech stimuli (Adachi et al. 2007; Gábor et al. 2019), the acoustic bases of familiar speaker recognition in dogs have never been explored.

In canines, the significance of recognizing the voices of different conspecific individuals (e.g., neighbor–stranger recognition in the territorial behavior of wolves (Coulter and Mech 1971)) is more obvious than that of heterospecific ones. Dogs (Molnár et al. 2009), wolves (Palacios et al. 2015; Balieiro and Monticelli 2019) and dingoes (Deaúx et al. 2016) can discriminate between conspecifics based on vocal cues. Statistical analyses revealed a wide variety of potential identity-diagnostic cues in vocalizations. These include fundamental frequency-, tonality-, sound- and spectral energy-related parameters (Yin and McCowan 2004; Molnár et al. 2008; Larrañaga et al. 2014). The single canine behavioral study looking for perceptually important voice identity markers in intraspecific contexts revealed an effect of frequency modulation pattern on voice identity discrimination in wolves (Palacios et al. 2015). Despite paucity of pertinent research, these findings show that canines can make use of vocal cues to infer vocalizer identities.

Humans are highly skilled in extracting identity information from speech from an early age. Fetuses (Kisilevsky et al. 2003) and newborns (Decasper and Fifer 1980) respond differentially to their mother’s speech, 7-month-old infants can discriminate between unfamiliar speakers talking in their native language (Johnson et al. 2011), and adults can remember both familiar and unfamiliar speakers for a long time with high accuracy (Papcun et al. 2005). A wide variety of speaker identity markers potentially suitable to facilitate these abilities were revealed by acoustic analyses. These include cues related to fundamental frequency (e.g., *f*_*0*_ mean, *f*_*0*_*-SD*), formants (e.g., formant dispersion: *dF*, or certain formant frequencies: *F5*) or noisiness (e.g., jitter: *ppj*, shimmer) (Baumann and Belin 2010). Perceptually important cues for voice identity recognition involve *f*_*0*_, *dF* and harmonicity (i.e., harmonics-to-noise ratio: *HNR*) (Belin et al. 2004; Latinus and Belin 2011; Latinus et al. 2013). That humans are sensitive to speaker identity cues, however, is not surprising, as the human perceptual system is tuned to a variety of cues in speech (e.g., Diehl et al. 2004).

Both efficient processing of speech content, and recognition of certain humans are important for companion dogs (Miklósi 2015). Selecting dogs to prefer humans resulted in close social relationships between family dogs and their owners (Hart 1995; Miklósi 2015). Living in the human social environment has made dogs highly responsive to speech. Indeed, dogs can process and rely on both non-linguistic (e.g., emotional valence) and linguistic (e.g., lexicality) cues in speech (Kaminski et al. 2004; Andics et al. 2014, 2016; Gábor et al. 2020). Regarding speaker information, dogs can differentiate between female and male voices (Ratcliffe and Reby 2014) and they can match their owner’s voice and face (Adachi et al. 2007). Recent research showed that dogs can distinguish between unfamiliar speakers both behaviorally (Root-Gutteridge et al. 2019) and neurally (Boros et al. 2020), and they can discriminate their owner’s live voice from that of an unfamiliar speaker of the same gender (Gábor et al. 2019). Further, in dogs, a secondary auditory brain region is more sensitive to the owner’s praise than to that of a familiar control person (Gábor et al. 2021). Although whether dogs use the same acoustic cues as humans to identify familiar speakers, is currently unknown, a better understanding of such difference or similarity would be informative regarding the extent to which dogs’ perceptual system is attuned to cues carried by human speech.

Hence, the aim of this study was to examine which, if any acoustic cues dogs rely on to discriminate their owner’s voice from other voices. We assumed that discrimination performance in dogs will be associated with the distance between speakers with regard to the acoustic cues used by dogs.

## Methods

### Subjects

We tested 28 family dogs, accompanied by their owners (50% male). To increase generalizability, dogs represented a variety of breeds: 19 purebreds of 14 breeds (2 Hungarian Vizslas, 2 Poodles, 2 Beagles, 2 Cocker spaniels, 2 American Staffordshire terriers, 1 Newfoundland dog, 1 Pointing Griffon, 1 Border collie, 1 Golden retriever, 1 Shetland sheepdog, 1 Malinois shepherd, 1 Bobtail, 1 Schnauzer, 1 Airedale terrier) and 9 mixed breed dogs (mean age ± SD in years: − 4.7 ± 2.2, range 1–8 years; 17 females and 11 males). There were 43 dogs in the original sample, but 14 (4 purebreds [1 Labrador retriever, 1 Boston terrier, 1 Shar-pei, 1 Puli dog] and 10 mixed breed dogs; 5 with female and 9 with male owners) were excluded after the training phase because they did not pass test criteria.

### Experimental setting

**Fig. 1:**
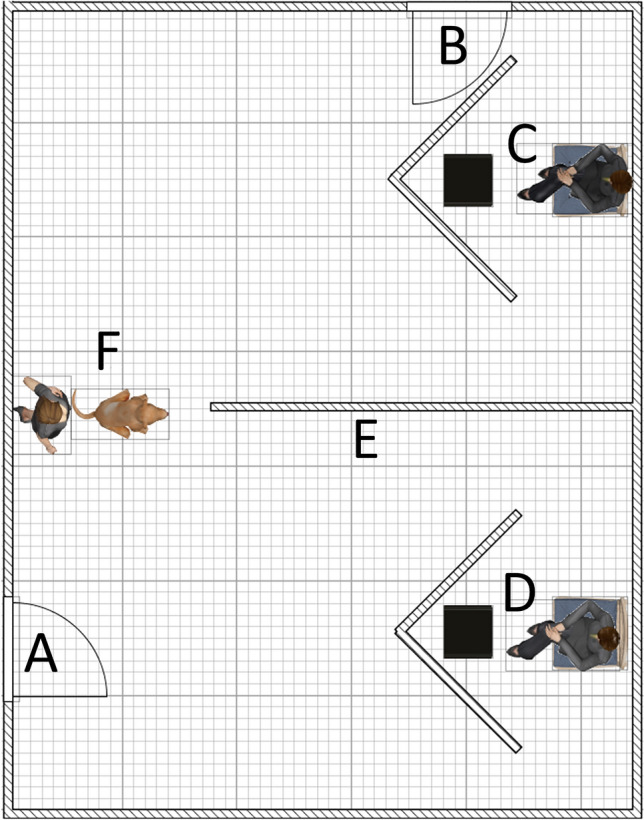
Illustration of the experimental setting. **A**, **B**: doors, **C**, **D**: location of the owner, Experimenter 1 and the loudspeakers; **E**: plastic wall; **F**: starting point which was the location of the dog and Experimenter 2 at the beginning of each trial. This Figure was prepared using SweetHome3D software developed by eTeks (http://www.sweethome3d.com/)

Experiments took place in a laboratory room (5.4 m × 6.3 m, Fig. 1) at the Department of Ethology, Eötvös Loránd University, Budapest, Hungary. The task for dogs was to find their owners, based solely on his/her voice. The lab room had two doors (Fig. 1A, B), two non-transparent screens placed in two corners of the room (the width of the screens’ two wings: 2 × 1.02 m, height: 1.25 m) and a plastic wall (Fig. 1E) placed in-between the screens. Owners and Experimenter 1 hid, and loudspeakers were also placed, behind the screens (Fig. 1C, D). The purpose of the wall was to ensure dogs have to make a decision regarding their owner’s location immediately after leaving the starting point (Fig. 1F).

In each trial, dogs were positioned at the starting point and speech stimuli from the owner and a control person were played. The starting point was positioned 3.64 m from the screens given prior findings indicating that dogs are barely able to find their hidden owners based on olfactory cues from 3 m (Polgár et al. 2016). Both screens and the wall were blue. The experiment consisted of 3 phases: a training, a test, and an olfaction control phase.

### Stimuli

In the training phase, first, the owner called the dog in live voice, then, depending on the trial, live neutral speech of either the owner or Experimenter 1 was used as stimulus. Calls started with the dog’s name and ended with commands the owners would typically use to call the dog (e.g., ‘*Suzy,* *come* *here*!’ [in Hungarian]). In the test and olfaction control phases, stimuli were pre-recorded neutral speech sentences from recipes (e.g., ‘*Wash* *the* *tomatoes* *and* *peppers* *in* *cold* *water.*’ [in Hungarian]) played through loudspeakers (training: 48 sentences, containing 6 – 11 words, mean word number ± SD = 8.1 ± 1.4; test and olfaction control: 28 sentences, containing 5 – 10 words, mean word number ± SD = 7.1 ± 1.2). Sentences played during the test and the olfaction control phases were randomly chosen and balanced (see below for details) across dogs and trials. Before the test phase, stimuli were recorded from owners and 14 control persons (50% male). Control persons were unfamiliar to the dogs. Stimuli were pre-recorded using a Zoom H4n handheld recorder and an Audio-Technica Pro 8HEx hyper-cardioid dynamic head microphone and were edited (cut and normalized to a unified volume) with Adobe Audition CC 2018 software. Thus, each spoken sentence was set to the same volume. During the test and the olfaction control phases, recipe sentences were played through loudspeakers (placed directly in front of the hiding persons, ~ 3.7 m far from the dog) at 68 dB volume (loudspeakers: Technics SB-M300M2; amplifiers: Technics se-a909s and Technics su-c909u). Praat software was used for acoustic analysis along a range of cues that have been reported to play an important role in the acoustic communication of either canines (Yin and McCowan 2004; Larrañaga et al. 2014) or humans (Baumann and Belin 2010; Latinus et al. 2013; Drozdova et al. 2017). Each spoken sentence was analyzed separately. For a detailed description of the acoustic features examined, see Table 1.

**Table 1:** Acoustic parameters

Variable	Description
Measures of fundamental frequency	
*f*_*0*_ mean	Mean fundamental frequency
*f*_*0*_ range	Range (*f*_*0*_ max—*f*_*0*_ min) of fundamental frequency
*f*_*0*_ change	Slope (*f*_*0*_ end—*f*_*0*_ st) of fundamental frequency
*f*_*0*_ sd	Standard deviation of fundamental frequency
*f*_*0*_ max	Maximum fundamental frequency
*f*_*0*_ min	Minimum fundamental frequency
*f*_*0*_ mnpozr	Relative position of minimum fundamental frequency (time of *f*_*0*_ min/call length)
*f*_*0*_ mxpozr	Relative position of maximum fundamental frequency (time of *f*_*0*_ max/call length)
*f*_*0*_ end	End fundamental frequency
*f*_*0*_ st	Start fundamental frequency
Measures of noisiness	
*ppj*	Jitter: periodicity of vocal fold vibration
*ppp*	Number of voice cycles
*ppm*	Mean number of voice cycles
*ent*	Wiener Entropy: uniformity of the spectrum
*HNR*	Harmonics-to-noise ratio: the degree of acoustic periodicity
*HNR* *SD*	Standard deviation of the HNR
*HNR* *max*	Maximum HNR
Measures of spectral energy	
*dF*	Formant dispersion: average frequency difference between the first five consecutive formants
CG	Center of gravity: average frequency in the spectrum
Dev Freq	Deviation frequency: standard deviation of the center of gravity in the spectrum
Energy Diff	Energy difference between 0–2000 and 2000–6000 Hz bands
Sk	Skewness of the spectrum
Kr	Kurtosis of the spectrum
cmoment	Non-normalized skewness of the spectrum
BEn	Band Energy: density of the spectrum between 2000 and 4000 Hz

### Experimental protocol

The experiment consisted of three phases without a break and in a fixed order: (1) training, (2) test (owner’s voice played form congruent side) and (3) olfaction control (owner’s voice played from incongruent side; Table 2). The task of dogs during the 3 phases was to follow their owner’s voice and approach one of the screens. Except at the beginning of the training phase (described later), dogs had to differentiate their owner’s voice from a set of control voices. Four humans participated in the experiment: the owner, Experimenter 1 who, at the same time as the owner, was hiding behind one of the screens (10 persons played this role), Experimenter 2, who accompanied dogs during the experiment (3 persons played this role), and Experimenter 3, who played the pre-recorded stimuli from a control room. The gender of the owner and Experimenter 1 always matched. Before the training phase (~ 5 min), the dog was allowed to explore the lab, including the area behind the screens. In the meantime, Experimenter 2 explained the experimental protocol to the owner. All phases consisted of a different number of trials (see later). At the beginning of each trial, Experimenter 2 led the dog on a leash into the lab through Door A and positioned it to the starting point (E), in front of herself, with the dog’s head facing toward the wall separating the two screens (Fig. 1). Then, Experimenter 2 indicated that stimulus presentation would begin, either by saying “OK” (in live-speech phases) or looking up (in recorded stimuli phases). During presentation of the stimuli, Experimenter 2 was standing behind the dog (thus out of its sight), looking straight ahead (never at the dog), to avoid inducing a Clever Hans effect (Pfungst 1911). At the end of each stimulus, Experimenter 2 said to the dog: ‘Go for it!’ in Hungarian. If the dog did not start moving toward either of the screens, Experimenter 2 slightly pushed the dog’s back toward the wall to motivate it to move. Earlier findings obtained in a similar setup show that, if the task is clear to the dog, then its’ performance cannot be influenced in two-way choice tasks by humans (even intentionally) (Hegedüs et al. 2013). Nevertheless, we paid extra attention to avoid potential Clever Hans effects. Experimenters participating in the study had experience with behavioral tests and were aware of the potentially biasing effects of their actions. Experimenter 2 was strictly instructed not to influence the dog’s choices, neither with his/her movements nor with the direction of his/her gaze. Further, the behavior of Experimenter 2 was always checked for any such instances of potential influence, by those scoring the videos; there was no evidence of a Clever Hans effect.

**Table 2:** Experimental protocol

Phase	No. of trials	Stimulus	Stimulus type	Speakers
1. Training	2	Naming and calling the dog	Live	Owner
	2	Neutral speech	Live	Owner
	Max 6	Neutral speech	Live	Owner vs Experimenter 1
2. Test	10	Neutral speech	Playback	Owner vs Control persons
3. Olfaction control	2	Neutral speech	Playback	Owner vs Control persons

This table shows the number of trials, stimuli, stimulus types and speakers involved in the different phases of the experiment

In case of a correct owner choice, the dog was rewarded. Both the owner and Experimenter 1 kept a bowl of food reward (sausage) with them. Additional details on how dogs’ choices were responded to are presented below. Following the response, the owner and Experimenter 1 went to the starting point, and Experimenter 2 instructed them where to hide for the next trial. After this, at the end of the trial, Experimenter 2 led the dog out of the room through Door A. One trial lasted around 2 min, and Experimenter 2 was outside with the dog for about 1 min between the trials. During this ~ 1-min period, the owner and Experimenter 1 took their positions behind the screens and the next trial started. Door B was never used during the experiments.

#### Training

The aim of the training phase was to familiarize dogs with the experimental conditions and to teach them, step-by-step, that their task was to choose their owner’s voice. The training consisted of three subphases (Table 2). First, in 2 trials, only the owner hid behind one of the screens (once on each side, C or D, Fig. 1) and called the dog. Second, in the next 2 trials, only the owner hid (again, once on each side), but this time, he/she read 2 neutral speech sentences aloud (with 4-s-long silent intervals between them). Third, in the next (maximum) 6 trials, the owner and Experimenter 1 both hid behind the screens and alternated reading 2–2 neutral speech sentences aloud. The two speakers were instructed to speak at a similar (clearly audible) volume. Correct owner-choices were reinforced by food and social reward (praise and pet) by the owner. If the dog made an incorrect choice (went to the screen hiding Experimenter 1), Experimenter 1 stood up and turned his/her back on the dog. In case of the first incorrect choice (if there was any), Experimenter 2 took the dog’s leash and led the dog to the screen hiding the owner. At this point, the owner rewarded the dog as if it chose correctly, to maintain its motivation. If the dog did not leave the starting point following stimulus presentation on more than one occasion, it was excluded from the experiment (*n* = 2). Similarly, if the dog was not able to find the owner on 3 consecutive occasions during Training trials 5–10, it was excluded (*n* = 13). If the dog reached test criteria (found its owner in 3 consecutive trials after trial 4) before the 10th trial, training was ended, and testing started. During the subphases of the training, hiding sides, who is the last speaker, and neutral speech sentences were pseudorandomized and balanced across trials, also, the owner hid behind the same screen on a maximum of 2 consecutive trials.

#### Test

The test phase followed the training phase (with no break between phases). During the 10 trials, both the owner and Experimenter 1 hid just like during the training, but this time pre-recorded neutral speech stimuli were played from the loudspeakers (Table 2). The owner’s voice came from behind the screen he/she was hiding behind and control voices came from behind the screen Experimenter 1 was hiding behind. In every trial only one control voice was used, but control voices differed across trials. Dogs were reinforced as in the training phase, but in the test phase, Experimenter 2 never helped them. Correct owner choices were scored as 1 and incorrect choices were scored as 0. The sum of these scores quantified choosing performance. Hiding sides, last speakers and neutral speech sentences were pseudorandomized and balanced across trials. In this phase, the owner hid behind the same screen on a maximum of 3 consecutive trials.

#### Olfaction control

In the 2-trial-long olfaction control phase, which followed the test phase (with no break between phases), hiding persons and voices were mismatched (Table 2). Thus, the owner’s voice came from behind the screen that hid Experimenter 1. Similarly, the control voice came from behind the screen that hid the owner. In the first trial, if the dog made a correct choice, that is, it went toward the owner’s voice and did not find her/him, Experimenter 1 behaved as if it was an incorrect choice, stood up and turned his/her back on the dog and the dog’s choice was scored as correct (= 1). If the dog made an incorrect choice, that is, it followed the control voice and found the owner, the owner behaved as if it was a correct choice, rewarded the dog, and the dog’s choice was scored as incorrect (= 0). After the 2nd trial, dogs were rewarded irrespective of their choice by both the owner and Experimenter 1. Hiding sides and last speakers were randomized and balanced across trials. As olfaction control trials were added later to the design, only 23 of the 28 dogs completed them.

### Data coding and analyses

Three behavioral variables were coded: (1) choosing success: a binomial score/trial, indicating whether or not the dog followed its owner’s voice, (2) looking time: the proportion of time (between first stimulus onset and the “Let’s go!” command) the dog spent with its head turned toward its owner’s voice and (3) choosing latency: the time spent between the “Let’s go” command and the dog’s front leg reaching the edge of a screen. Data were video recorded, and videos were scored using the Solomon Coder software (https://solomon.andraspeter.com/). Four cameras were placed in the ceiling, in different locations to permit visibility of the entire room. One camera recorded the dog from the front, allowing for precise estimates of looking time. The plastic wall placed between the screens—and thus at the midline of the dog’s head—further aided measurement of looking time. Data analysis was performed using Microsoft Excel 2019, R statistical environment (https://www.r-project.org/) and RStudio software (https://rstudio.com/). To determine which acoustic features dogs relied on most to discriminate between individuals, linear discriminant analyses (LDA, MASS package, https://cran.r-project.org/web/packages/MASS/index.html) (cf. Tooze et al. 1990) were conducted, with pre-defined speakers (owners and control persons) as grouping variables. All acoustic parameters listed in Table 1 were included in LDA models, except for that did not meet criteria for ‘independent variables’ based on Pearson correlation analyses (*f*_*0*_ *st*, *f*_*0*_ *range*, *f*_*0*_ *max*, *DevFreq*, *ppm*, *ppp*, *kurtosis*, *energydiff*). Acoustic distance between speakers was calculated separately for each trial using relevant LDA output variables. Acoustic distance was indexed by the absolute value of the difference between the two speakers’ values on a given parameter. These distance values were used as covariates in subsequent analyses aimed at examining the association between acoustic parameters and dogs’ voice discrimination ability.

To examine, first, whether dogs chose their owner’s voice more often than control voices during either test or olfaction control trials and second, which fixed factors (design parameters: trial, owner speaks last, owner’s hiding side, gender match of speakers) affected choosing success, a binomial generalized linear mixed model (GzLMM) was conducted. Here and in subsequent analyses, backwards elimination was used; fixed factors that were not associated (at least at the tendency level) with the dependent variables were removed stepwise. To examine whether there was a side or a last speaker effect on choosing success, two intercept only binomial GzLMMs were conducted. To test whether choosing latency and looking time were associated with choosing success, a binomial GzLMM was ran. To identify which acoustic cues affected looking time, a GLMM involving acoustic distance variables (along dimensions of *f*_*0*_ *mean,* *f*_*0*_ *SD,* *dF,* *HNR,* *entropy,* *ppj*) as covariates, and owner and control person gender as a binary fixed factor (same/different), was conducted. The two-way interactions between acoustic distance parameters and gender were also included in initial models. Dog and unfamiliar speaker identity were included as random factors in all models, except in cases where speaker identity explained no variance in the model and was thus omitted (intercept only models, looking time).

To examine whether the voices of the owners of excluded dogs differ from those of the owners of included dogs, or from human voices in general with regard to acoustic features implicated in the voice discrimination ability of dogs (defined as per the results of LDA analyses), a GLMM was conducted. Speaker type (owners of excluded and included dogs, and control persons) and gender (female or male) were included as three- and two-level fixed factors. An interaction effect between speaker type and gender was tested using a Likelihood Ratio (LR) test.

## Results

### Choosing success in test and olfaction control phases

**Fig. 2:**
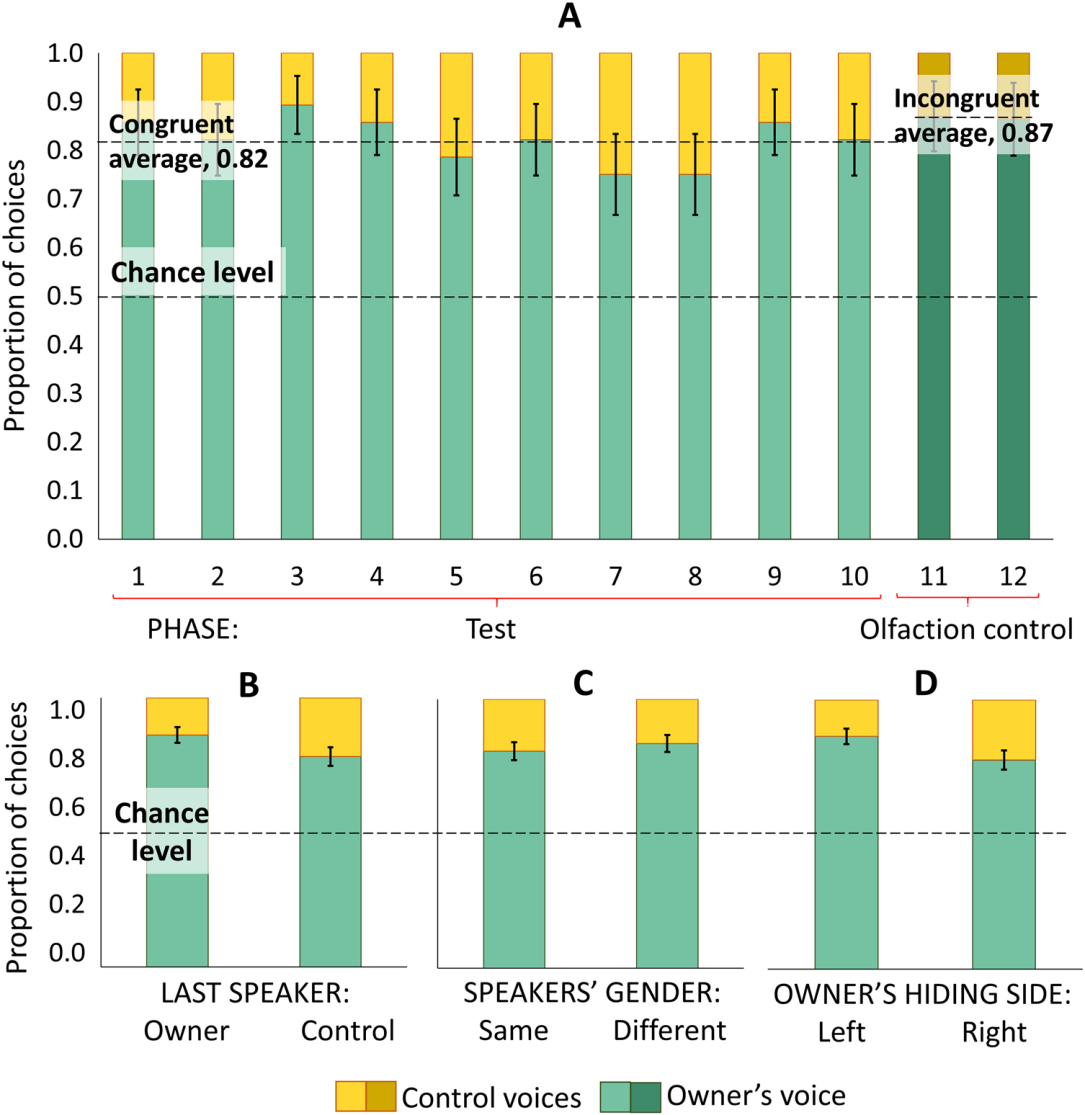
Proportion of owner and control voice choices. The figure shows the proportion of owner (green) and control voice (orange) choices per trial (**A**) and depending on the owner’s hiding side (**B**), the last speaker within trials (**C**), and the speakers’ gender match (**D**). Error bars represent SEM. Test: *N* = 28, olfaction control: *N* = 23

The intercept only binomial GzLMMs investigating dogs’ choosing success revealed that dogs chose their owner’s voice more often than the control voices during both the test and the olfaction control phases (Fig. 2A, Table 3). Wilcoxon signed-rank test revealed no difference in choosing success between the test and the olfaction control phases (*Z* = 0.000, *p* = 1.000).

**Table 3:** Dogs’ choosing success during the test and the olfaction control phases

Phase	Dependent Variable	Proportion of correct choices	Odds ratio	Estimate	SEM	*z*	*p*
Test	Choosing success	0.82	4.957	1.601	0.197	8.110	< 0.001
Olfaction control		0.87	7.385 × 10^12^	29.630	12.498	2.371	0.018

The table shows the results of the intercept only binomial GzLMMs. *SEM* standard error of mean. Test: *N* = 28, olfaction control: *N* = 23

### Effects of design parameters

The binomial GzLMM on dogs’ choosing success revealed no effect of trial number (Fig. 2A), speaker order (Fig. 2B), or gender match (Fig. 2C). Dogs chose their owner’s voice more often if he/she hid behind the left-sided screen (Fig. 2D) (Table 4). The intercept only binomial GzLMMs revealed no general side bias (odds ratio = 0.815, est. = -0.205, SEM = 0.134, *z* = − 1.535, *p* = 0.125) or last speaker bias (odds ratio = 1.121, est. = 0.114, SEM = 0.120, *z* = 0.956, *p* = 0.339) association with choosing success.

**Table 4:** Acoustic and design parameter effects on dogs’ choosing success

Dependent variable	Fixed effects	Estimate	SEM	*z*	*p*
Choosing success	Owner’s side: left	0.688	0.330	2.086	0.037
	Last speaker: owner	0.630	0.330	1.912	0.056

The table shows results of the binomial GzLMM on choosing success within the test trials. *SEM* standard error of mean. *N* = 28

### Acoustic discriminability of voice identities

The average discrimination success of the LDA was 88.2% showing high individual discriminability of the speakers. Based on discriminant function loadings, 6 acoustic parameters: *f*_*0*_ *mean,* *entropy,* *HNR,* *f*_*0*_ *SD,* *dF* and *ppj* (in this order) contributed to discrimination (having high loading [above 1.3 in absolute value] on at least one discriminant function. See Supplementary Information, Table S1).

### Relation of behavioral variables

**Fig. 3:**
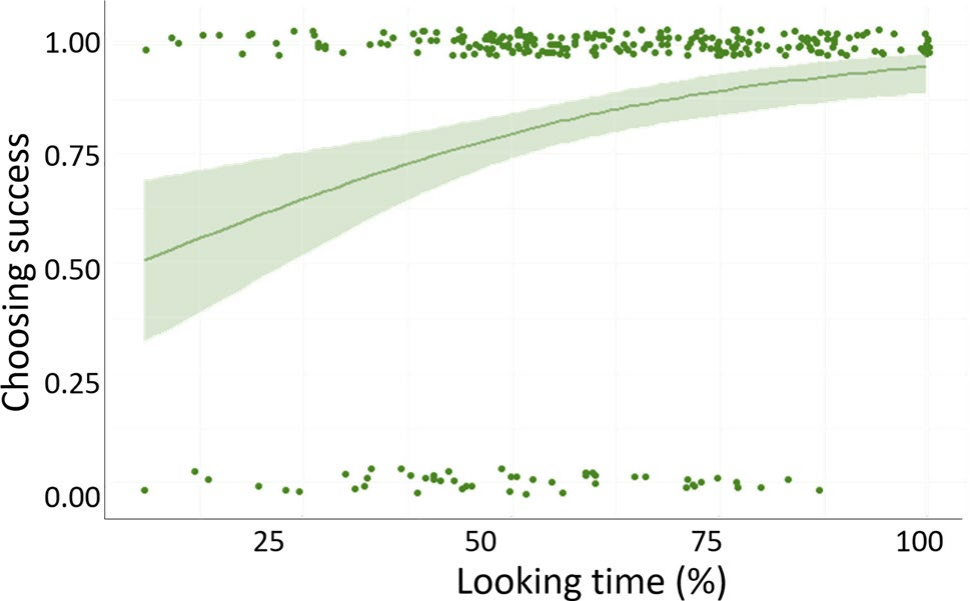
Positive association between choosing success and looking time. X-axis shows the proportion of time dogs spent looking toward the screen corresponding to their owner’s voice during stimulus presentations. Y-axis represents the proportion of correct (owner’s voice) choices. Each dot represents the results of one trial, so each trial of every dog tested is displayed. *N* = 28

The first binomial GzLMM revealed a positive effect of looking time, but no effect of choosing latency on choosing success (Table 5). That is, the longer the dogs looked in the direction of the owner’s voice the more likely they chose correctly (Fig. 3). As there was no association between choosing success and choosing latency, we assumed that choosing latency as measured here is not a reliable predictor of choice difficulty and thus excluded it from further analyses.

**Table 5:** Relation between behavioral variables

Dependent Variable	Fixed effects	Estimate	SEM	z	p
Choosing success	Looking time	0.031	0.009	3.648	< 0.001
	Choosing latency	− 0.130	0.069	− 1.809	0.059

Effects of choosing latency and looking time on dogs’ choosing success revealed by binomial GzLMM using choosing success as a dependent variable. *SEM* standard error of mean. *N* = 28

### Effects of speakers’ acoustic distance on looking time

**Fig. 4:**
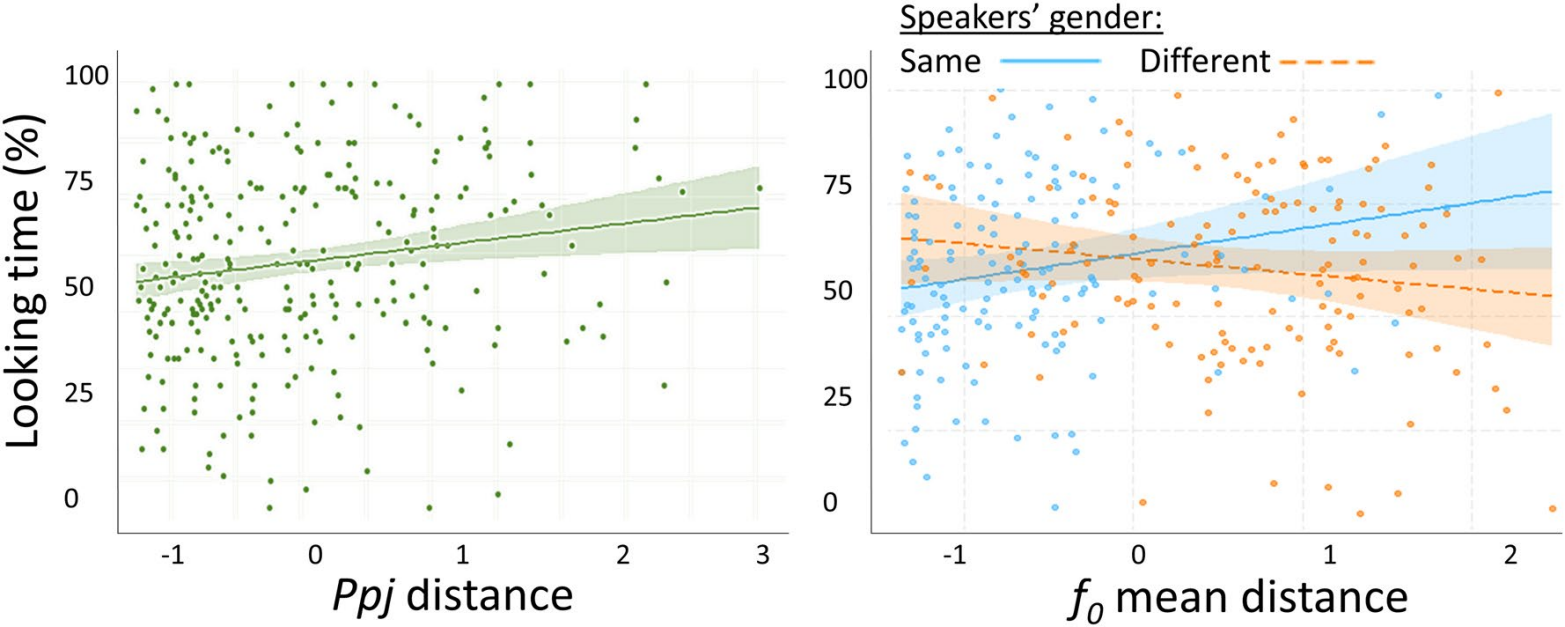
Effect of speakers’ acoustic distance on looking time. Association between looking time and jitter (*ppj*) speaker distance (left) and gender match by fundamental frequency (*f*_*0*_) mean speaker distance (right). *Ppj* and *f*_*0*_ *mean* distances are represented by z scores. Each dot represents the results of one trial, so each trial of every dog tested is displayed. *N* = 28

The GLMM investigating the effects of speakers’ acoustic distance on looking time revealed *f*_*0*_ *mean*, *ppj* and *f*_*0*_ mean by gender match effects (Table 6). More specifically, the larger the *f*_*0*_ *mean* and *ppj* distance between speakers, the greater the proportion of time dogs spent looking toward their owner’s voice during stimulus presentation. Post hoc tests indicated a positive effect of speaker *f*_*0*_ *mean* distance when speaker gender was the same (same gender: estimation = 5.613, SE = 2.775, *t* = 2.023, *p* = 0.044; different gender: estimation = − 3.343, SE = 2.507, *t* = − 1.334, *p* = 0.184) (Fig. 4).

**Table 6:** Effect of speakers’ acoustic distance on looking time

Dependent variable	Fixed effects	Estimation	SE	*t*	df	*p*
Looking time	Gender match	− 1.107	3.227	− 0.343	260.814	0.732
	*f*_*0*_ *mean* distance	5.613	2.775	2.023	207.948	0.044
	*Ppj* distance	4.010	1.729	2.319	258.586	0.021
	*f*_*0*_ *mean* distance × Gender match	− 8.956	3.885	− 2.305	135.823	0.023

Results of the GLMM investigating the effect of speakers’ acoustic distance on looking time. *f*_*0*_: fundamental frequency, *ppj* jitter. *N* = 28

We found no systematic *f*_*0*_ *mean* and *jitter* differences between either owner and control voices or owner voices of excluded and included dogs within female and male speakers (LR tests, F0 mean GLMM: *F* = 2.030, df = 2, *p* = 0.141; jitter GzLMM with Gaussian distribution and log link: 2 = 0.103, df = 2, *p* = 0.950). This suggests that excluded dogs did not fail the training phase due to acoustic extremities in their owner’s voice.

## Discussion

In this study, we presented evidence that dogs can use person identity information in the human voice to discriminate their owner’s voice from other speakers’ voices, and we identified two perceptually important acoustic markers (mean fundamental frequency: *f*_*0*_ *mean*, jitter: *ppj*) supporting this ability. In addition, we developed an experimental paradigm suitable to collect many active responses per dog to pre-recorded auditory stimuli in a single session.

Dogs’ high choosing success rate, their ability to discriminate their owner’s voice from a variety of control voices, and the fact that dogs’ choices were not confounded by either olfactory cues or speaker order indicate that dogs can reliably use identity cues carried by speech. Our findings on cues that contributed to acoustic discriminability of speaker identities are consistent with the results of other studies (Baumann and Belin 2010). Specifically, our results suggest that *f*_*0*_ *mean*, entropy (*ent*), harmonics-to-noise ratio (*HNR*), standard deviation of fundamental frequency (*f*_*0*_ *SD*), formant dispersion (*dF*) and *jitter* (*ppj*) are potentially implicated in speaker recognition. To recognize voices, however, individuals do not necessarily use the most pronounced acoustic markers of identity. Instead, individual recognition by auditory means can be achieved in different ways, probably depending on ecological pressures and evolutionary history (Kriengwatana et al. 2014). Thus, different species, such as humans and dogs, may not use the same identity cues to separate speakers.

The positive association between looking time and speaker acoustic distance in *f*_*0*_ *mean* and *jitter* indicates that these parameters serve as perceptually important owner voice identity cues for dogs. In general, the larger the *f*_*0*_ *mean* and the *jitter* distance, the easier it is for dogs to choose their owner’s voice. This association, however, is not as straightforward in case of *f*_*0*_ *mean*, as it affected looking time only when speakers were the same gender, whereas the pronounced *f*_*0*_ difference naturally discriminating genders (Gelfer and Mikos 2005) did not facilitate decisions. Therefore, it seems that there is a ceiling effect in *f*_*0*_ *mean* differences and looking behavior does not vary above such plateau: in case of robust *f*_*0*_ *mean* differences, dogs can successfully choose their owners’ voice. Below this threshold, however, the looking time of dogs depends on *f*_*0*_ *mean* speaker difference, indicating that the perceptual system of dogs may be less sensitive to slight *f*_*0*_ changes. Our finding that *f*_*0*_ is a primary parameter for differentiating between vocalizers is consistent with the results of studies conducted with a number of species (e.g., Aubin et al. 2000; Charrier et al. 2002), including humans (Lavner et al. 2001; Latinus et al. 2013) and canines (Palacios et al. 2015). Jitter also contributes to the acoustic discriminability of speakers (Farrús et al. 2007; Farrús and Hernando 2009; Baumann and Belin 2010). Although no studies have investigated whether dogs use jitter to separate canine voice identities, it has been reported that a larger jitter may increase dogs’ attention (Lehoczki et al. 2019), suggesting that variation in this acoustic cue has behavioral relevance. Although this study investigated the acoustic cues dogs rely on to identify their owner’s voice specifically, there is no reason to assume systematic acoustic differences between owner voices and human voices in general. The lack of *f*_*0*_ *mean* and *jitter* differences between owner and control voices indicates that owner voices provide a representative sampling of human voices in the acoustic space.

Whereas *dF* and *HNR* serve as perceptually important identity-diagnostic cues for humans (Belin et al. 2004; Latinus and Belin 2011; Latinus et al. 2013), we found no evidence that dogs use these parameters to identify their owner’s voice. We note that dogs do make use of both *dF* and *HNR* cues in conspecific vocalizations. For example, dogs can use *dF* in growls of conspecifics as cues for size (Faragó et al. 2010; Taylor et al. 2011), and noisier puppy calls lead to faster orientation to the vocalizer (Lehoczki et al. 2020). Acoustic analysis suggests that *HNR* contributes to acoustic discriminability of canine voice identities (Larrañaga et al. 2014). The perceptual relevance of *dF* and *HNR* in vocalizer identity discrimination has, however, never been investigated in canines in either conspecific or heterospecific contexts. According to the above studies, dogs can sense and rely on *dF* and *HNR* variations, but the present results indicate that they may not use these parameters to recognize familiar speakers. There are different explanations for this result. First, vocalization-general voice identity markers (e.g., *f*_*0*_) that are part of the voice discrimination repertoire of many species may, in and of themselves, be sufficient for dogs to identify their owner’s voice. Thus, although it is clearly important for dogs to recognize certain humans, there might not have been selection pressure on the involvement of additional acoustic parameters into dogs’ speaker discrimination ability. Second, in contrast to human studies (e.g., Latinus et al. 2013), we applied whole sentences instead of single phonemes or words to maximize stimulus naturalness. Due to the acoustic richness and variability of our stimuli, *f*_*0*_ *mean* could have been enough for dogs to choose the owner’s voice, obviating need to also rely on other cues. Furthermore, use of whole sentences allowed for potential natural biases to be explored. We further note here that when listening to longer speech segments, humans have been reported to also use emotional prosodic cues for speaker recognition besides low level cues (e.g., Xu and Armony 2021). Here we used emotionally neutral (recipe) sentences, so we did not test whether dogs could also use emotional prosodic cues for speaker recognition.

Due to dogs’ high owner voice preference, choosing success did not serve as a variable sufficiently sensitive to investigate the perceptual importance of specific acoustic cues of speaker identity. Thus, we had to examine the effect of acoustic variables along a more sensitive parameter. The positive association between looking time and choosing success suggests that when dogs decided about the location of the owner’s voice, they looked toward it. We can thus assume that the longer it took for dogs to first look in the direction of the owner’s voice, the harder the choice was. The voices of the speakers always followed each other in an alternating manner, which could influence looking time, because dogs could decide about the owner’s voice location earlier in case the owner spoke first in the trial. To control for this, the first speaker’s identity (owner/control voice) was balanced and pseudorandomized. Unlike looking time, choosing latency was not significantly associated with choosing success, and thus did not serve as a reliable variable to investigate which acoustic cues dogs use to separate owner and control voices. This is probably because, due to design restrictions, we could only measure choosing latency from the end of stimulus presentations. By this time, several seconds had passed, and the dogs had probably already made their decision.

In this study, we presented an experimental setting for auditory tests with dogs that combines the flexibility of using pre-recorded stimuli with the motivational advantage provided by the owner’s involvement. To behaviorally measure which acoustic parameters influence voice discrimination ability, adult humans are usually asked to judge speaker similarity (Lavner et al. 2001; Baumann and Belin 2010; Latinus and Belin 2011). In contrast, the application of a paradigm requiring active responses is cumbersome in case of subjects with limited linguistic skills, and thus the collection of a sufficient amount of data per individual is typically problematic (Caron et al. 1977; Ono et al. 2015). Our method is, however, based on active responses and is suitable to collect a large amount of data per dog in a single session. During the test, dogs’ performance did not deteriorate with repeated trials, suggesting that their attention was sufficiently maintained with food rewards and natural proximity seeking with the owner. Furthermore, after some experience with the experimental setting, most dogs could stably rely on playback sounds despite other research suggesting that dogs’ performance decreases when live-speech stimuli are switched to recordings (Pongrácz et al. 2003) or dehumanized computer commands (Gibson et al. 2014). The use of recordings in this design allows for a detailed acoustic analysis.

Dogs chose their owner’s voice with a higher chance if it came from the left side. Consistent with this finding, Karl et al. (2020) found behavioral preference for the owner if it appeared on the left. The effect of the owner’s side found in these studies might reflect right-hemispheric bias for either more familiar or more emotional stimuli (Siniscalchi et al. 2017). The fact that dogs were not more likely to choose one side or the other in general, strengthens that the above effect was related to the owner’s identity, and thus further supports the perceptual lateralization behind this bias related to more emotional stimuli.

Overall, our findings show that dogs can identify their owner based on vocal cues of identity. We also revealed perceptually important acoustic parameters dogs use to discriminate their owner’s voice from unfamiliar voices. This is the first study to reveal perceptually important voice identity markers used to discriminate between voices of heterospecific individuals. Our findings indicate that dogs use some but probably not all acoustic cues that humans use to identify familiar speakers. Although dogs can detect fine changes in speech, their perceptual system may not be fully attuned to identity-diagnostic cues in the human voice.

## Supplementary Information

Below is the link to the electronic supplementary material.

Supplementary file1 (PDF 337 KB)

Supplementary file2 (RAR 18,972 KB)

## Data Availability

The data that support the findings of this study are available upon request from the corresponding author (AG: annagabor33@gmail.com).
