# Supplementary material for: The acoustic bases of human voice identity processing in dogs
Source: Anim Cogn. 2022 Feb 10;25(4):905–16. doi: 10.1007/s10071-022-01601-z (PMC9334438; doi:10.1007/s10071-022-01601-z)
Supplement: Supplementary file 1 — Supplementary file1 (PDF 337 KB) [file 10071_2022_1601_MOESM1_ESM.pdf]

Supplementary Information

The acoustic bases of speaker recognition in dogs

Anna Gábor[1,2], Noémi Kaszás[2], Tamás Faragó[2], Paula Pérez Fraga[1,2], Melinda Lovas[2], Attila Andics[1,2]

[1]MTA-ELTE 'Lendület' Neuroethology of Communication Research Group, Hungarian Academy of Sciences - Eötvös Loránd University, 1/C Pázmány Péter sétány, Budapest, 1117-Hungary

[2]Department of Ethology, Eötvös Loránd University, 1/C Pázmány Péter sétány, Budapest, 1117-Hungary

Table S1. Results of the LDA investigating the acoustic discriminability of voice identities.

	LD 1	LD 2	LD 3	LD 4	LD 5	LD 6	LD 7	LD 8	LD 9	LD 10	LD 11	LD 12	LD 13	LD 14	LD 15	LD 16	LD 17
f0 mean	**5.35**	-0.51	0.74	0.37	-0.75	**2.53**	-0.79	-0.32	1.04	**-1.72**	1.06	-0.30	0.38	1.19	1.11	0.11	0.59
ent	0.49	**1.64**	**-1.99**	-0.64	-0.04	-0.08	0.04	-0.07	0.07	0.15	0.10	-0.23	0.28	-0.03	0.10	-0.01	-0.17
dF	0.35	**-1.43**	**-1.48**	**-1.34**	0.35	0.29	0.03	-0.25	-0.11	0.08	0.06	0.03	0.23	-0.01	-0.10	-0.11	0.13
HNR	-0.31	-0.14	-1.18	**1.40**	-0.66	**-1.73**	-0.15	**-1.83**	0.78	0.58	-0.35	-0.51	-0.28	0.00	-0.59	0.24	0.53
f0 SD	0.84	0.37	0.51	-0.65	**1.61**	**-1.86**	0.15	-0.09	-0.57	0.45	-0.76	-0.22	0.06	-0.01	-0.18	-0.34	0.12
cmoment	0.19	-0.21	0.31	-0.35	-1.20	-0.91	-0.10	0.11	0.55	-0.15	-0.61	-0.04	-0.34	0.01	0.11	-0.09	0.07
SK	0.06	-0.21	-0.05	0.49	-0.54	-0.86	-0.37	0.80	-0.75	-0.18	0.83	-0.01	0.47	-0.14	0.28	-0.02	-0.08
CG	-0.13	-0.18	0.23	0.27	-0.34	-0.45	0.25	-0.29	0.00	0.59	0.25	0.83	0.70	-0.03	-0.04	0.49	0.43
f0 min	-0.02	0.17	0.39	-0.26	0.19	0.43	-0.48	0.30	-0.69	0.25	-0.82	-0.04	0.43	-0.24	0.30	-0.57	0.22
BEn	0.13	-0.08	-0.05	-0.10	-0.63	-0.35	0.08	0.08	-0.15	0.56	0.80	-0.68	0.17	-0.11	-0.12	-0.62	0.06
ppj	-0.36	0.05	-0.16	0.29	0.25	-0.21	**-1.32**	-0.41	**1.38**	0.50	0.47	-0.12	0.45	0.02	0.20	-0.17	0.30
HNR SD	-0.19	-0.16	-0.26	0.41	0.52	0.17	0.66	1.07	0.66	1.00	-0.30	0.26	0.38	-0.10	0.17	0.12	-0.02
f0 end	0.30	0.03	0.06	-0.27	0.11	-0.16	-0.53	0.15	-0.17	0.88	0.15	0.43	-0.65	-0.98	-0.77	0.69	-0.93
HNR max	0.09	0.08	-0.05	-0.01	-0.07	-0.16	0.07	0.13	0.11	0.01	0.33	0.86	0.17	0.26	-0.13	-0.75	-0.36
f0 mnpozr	-0.05	0.00	-0.03	0.05	0.05	-0.02	0.13	-0.32	0.00	0.26	0.12	0.08	-0.15	-0.08	0.88	-0.02	-0.47
f0 change	-0.20	-0.07	0.06	-0.04	0.04	0.02	-0.01	-0.14	-0.22	0.00	-0.26	-0.21	0.21	**1.30**	0.44	-0.01	0.02
f0 mxpozr	-0.07	-0.02	-0.13	-0.04	-0.02	-0.01	-0.05	0.23	-0.11	0.17	0.16	0.33	-0.68	0.00	0.17	-0.07	0.66

For an explanation of the abbreviations of acoustic parameters see Table 1. Red: positive values, blue: negative values. The more intense colour indicates a higher loading value. High loading factors are marked with bold. LD = discriminant factor. N=28.
